# Characterization of Fungal Endophytes Isolated from the Metal Hyperaccumulator Plant *Vachellia farnesiana* Growing in Mine Tailings

**DOI:** 10.3390/microorganisms8020226

**Published:** 2020-02-08

**Authors:** Giovanni Salazar-Ramírez, Rosario del Carmen Flores-Vallejo, Julio César Rivera-Leyva, Efraín Tovar-Sánchez, Ayixon Sánchez-Reyes, Julio Mena-Portales, María del Rayo Sánchez-Carbente, María Fernanda Gaitán-Rodríguez, Ramón Alberto Batista-García, María Luisa Villarreal, Patricia Mussali-Galante, Jorge Luis Folch-Mallol

**Affiliations:** 1 Centro de Investigación en Biotecnología; Universidad Autónoma del Estado de Morelos, Cuernavaca 62210, Morelos, Mexico; giovanudo@hotmail.com (G.S.-R.); rosariofvallejo@gmail.com (R.d.C.F.-V.); maria.sanchez@uaem.mx (M.d.R.S.-C.); mf.gaitan.rodriguez@gmail.com (M.F.G.-R.); luisav@uaem.mx (M.L.V.); 2 Facultad de Farmacia, Universidad Autónoma del Estado de Morelos, Cuernavaca 62210, Morelos, Mexico; julio.rivera@uaem.mx; 3 Centro de Investigación en Biodiversidad y Conservación, Universidad Autónoma del Estado de Morelos, Cuernavaca 62210, Morelos, Mexico; efrain_tovar@uaem.mx; 4 Cátedras CONACyT-Instituto de Biotecnología; Universidad Nacional Autónoma de México, Cuernavaca 62210, Morelos, Mexico; ayixon.sanchez@mail.ibt.unam.mx; 5 Instituto de Ecología y Sistemática, Carretera Varona 11835 e/ Oriente y Lindero, Capdevila, Boyeros, 11900 La Habana 19, Cuba; 6 Centro de Investigación en Dinámica Celular; Universidad Autónoma del Estado de Morelos, Cuernavaca 62210, Morelos, Mexico; rabg@uaem.mx

**Keywords:** Bioremediation, endophytes, heavy metals, *Vachellia farnesiana*, metal solubilization, organic acids, antioxidant secondary metabolites

## Abstract

Heavy metal pollution has become an environmental and health problem worldwide. With the aim of finding novel strategies for metal bioremediation, endophytic fungi from the heavy metal hyperaccumulator plant *Vachellia farnesiana* were isolated and characterized. The plants were growing in mine tailings, rich in Zn, Pb, and Cu. Morphological and phylogenetic analyses indicated that the fungal strains belonged to *Neocosmospora* and *Aspergillus* genera. The *Neocosmospora* isolate belongs to the *Fusarium solani* species complex (FSSC) that groups phytopathogen species. However, in this case the plants from which it was isolated did not show any signs of disease. Both fungal strains were able to remove significant amounts of heavy metals from liquid cultures, either in a mixture of the three metals or each metal in a single culture. In response to lead exposure, the *Neocosmospora* sp. strain secreted specific novel phenolic compounds other than anthraquinones or naphtoquinones, which have been described in similar situations. The *Aspergillus* sp. dropped the pH in the medium. High-performance liquid chromatography determinations indicated that this strain secreted mainly glutamic acid in response to lead, a novel mechanism, which has not been reported elsewhere. Malic and succinic acids were also produced in response to lead exposure. Possibly, glutamic and succinic acids (synthesized in the Krebs cycle) can be used to cope with metal toxicity due to the plant providing photosynthates to the fungus. These fungi showed the potential to be used for bioremediation or restoration of metal-polluted environments.

## 1. Introduction

Mining is a necessary but very polluting activity. Usually, its target consists of recovering a single type of metal from mines, leaving behind rocks with a relative high content of other heavy metals, which pose a threat to living organisms and overall environmental health [1]. As a consequence of mining activities, millions of tons of mine wastes are left in the environment, which are known as mine tailings and may contain different heavy metals or semimetals [2]. Mine tailings have become a health and environmental issue worldwide, and investigations that aim to solve this matter must be looked after.

Toxicity of heavy metals lies on the fact that they can interact in several biochemical pathways in the cell. Probably, the most important and common mechanism of toxicity is the generation of Reactive Oxygen Species (ROS), which in turn affect proteins and DNA, rendering the first inactive and causing mutations in the latter [3]. Frequently, heavy metals interact directly with proteins (including enzymes, transcription factors, or structural proteins), either directly in the catalytic site or in other parts, altering the three-dimensional structure and thus, inactivating the protein [4].

There are two main mechanisms to reduce heavy metals toxicity and both involve redox reactions. One mechanism consists of rendering the metal insoluble, so it is not bioavailable. However, in this case the metals are not extracted and eventually can change their redox state to become again soluble and, therefore, toxic for living organisms. The other mechanism consists of reducing metals to soluble forms that can be taken by living organisms, and some of these can cope with the toxicity in various ways (see below) [5]. The mechanisms for treating heavy metals-polluted soils might be achieved chemically, physically, or biologically (bioremediation). Physical and chemical strategies for remediation of contaminated soils are costly and scarcely efficient. Regarding bioremediation, plants have been the most extensively studied biological species to remediate soils polluted with heavy metals [6,7], but currently the use of microorganisms such as bacteria and fungi has attracted attention because of the discovery of multiple benefits on the bioremediation processes from the plant–microorganism association [8].

Heavy metals are ecologically very difficult to handle since they are not “degradable” as other compounds such as hydrocarbons, antibiotics, or pesticides, therefore, their persistence in the biosphere is almost permanent (for decades or even centuries) and they tend to bioaccumulate in many organisms, resulting in their biomagnification along the food chains. As mentioned above, some microorganisms have strategies to cope with heavy metal toxicity. In particular, fungi are good candidates for the bioremediation of heavy metals since they possess a number of mechanisms for metal detoxification [9,10]. They can adsorb metals in their cell wall, which is composed primarily of chitin and β-glucans that are rich in hydroxyl groups, enabling them to chelate metal ion. Also, the amino group of chitin can coordinate the metals, immobilizing them [11]. Fungi have vacuoles very similar to those of plants in which they can accumulate metals, preventing them from interfering with the cell’s cytoplasmic metabolism [12]. Moreover, they have biochemical mechanisms to cope with the oxidative stress generated by heavy metals, such as the synthesis of glutathione, thioredoxins, and metallothioneins, all of which are capable of sequestering heavy metals, making them innocuous to the cell [13,14]. Finally, fungi have been proved to secrete organic acids that interact with heavy metals, forming insoluble salts which render the metals nontoxic. Hence, studies assessing interactions between hyperaccumulator plants and their endophytes are necessary in order to propose them as a tool for the enhancement of bioremediation strategies when using hyperaccumulator plants.

In our group, we are interested in the restoration of mine tailings and as a model we have studied a site in Huautla, Morelos State, Mexico (18°. 43’ 33’’ N–99°. 03’ 33’’ W). The works by Cervantes-Ramírez et al. [2] characterized the type and concentration of heavy metals in one of three existing mine tailings that were exploited during the XVIII and XIX centuries for the extraction of lead, silver, and zinc [15]. The concentrations of Pb, Zn, and Cu were around 0.2 g/kg; 2.4 g/kg, and 0.03 g/kg of soil, respectively.

In Huautla, metal ores were exploited, resulting in more than 780,000 tons of mine wastes rich in Pb, As, Cu, Zn, and Cd. Recent studies have documented metals in drinking water and negative effects on human health [16], detrimental effects of metal bioaccumulation on small mammal species [17,18], and bioaccumulation in the surrounding biota: specifically, in hyperaccumulator plant species established on the mine tailings. Among these, *Vachellia farnesiana* (L.) Wight & Arn, a leguminous tree and one of the most dominant species in the zone, grows in these mine tailings and bioaccumulates preferentially Pb, Cu, and Zn in leaves (Pb = 0.149 g/Kg; Zn = 2.495 g/Kg; Cu = 0.0815 g/Kg [19]. Hence, we decided to look for the presence of fungal endophytes in *V. farnesiana* roots growing in the mine tailing and explore their bioremediation capabilities with regard to these three heavy metals.

## 2. Materials and Methods

### 2.1. Collection Site

Huautla mine tailing (18°26′ 36.37′′ N–99°01′26.71′′ W) is located at the southern region of Morelos state in the municipality of Tlaquiltenango, at an altitude of 974 m (INEGI, 2009). Huautla is located inside a protected natural reserve known as the REBIOSH [17]. This tailing has been previously characterized in terms of its physical-chemical properties and metal content. It is enclosed by deciduous forest [20], has a pH of 8.2, a cation exchange capacity of 30.1 cmol (+)/kg, where the predominant particulate size is <45 μm (44.2%), being this fraction where the highest metal concentrations are contained [21,22]. Metal solubility percentages of this mine tailing corresponded to Pb, 58.6%, Cu, 80.6%, and Zn, 32.1%. [17].

### 2.2. Vachellia farnesiana Sampling and Root Collection

We chose four healthy looking *V. farnesiana* individuals with approximately 1.5 m of height from the Huautla mine tailing. The collection took place in the summer of 2017, before the raining season started. Each individual was extracted from the mine tailing, and their adventitious roots were cut in 10 cm long pieces, then, root pieces were transported to the laboratory inside an ice box under darkness at 4 °C and immediately processed. Samples of the individuals were identified as *Vachellia farnesiana* (L.) Wight & Arn. (*syn. Acacia farnesiana* (L.) Willd) at the HUMO herbarium (Herbarium of the University of MOrelos, Centro de Investigación en Biodiversidad y Conservación, UAEM) and deposited with the following voucher numbers: 25048 and 25049.

### 2.3. Fungal Endophyte Isolation

A surface sterilization procedure was carried out to ensure the isolates were endophytes and not rhizospheric fungi [23]. The complete roots were first washed with tap water for 10 minutes and then washed for five more minutes with sterile double-distilled water. Then, inside a laminar flow hood, the roots were submerged for three minutes in a 70% *v/v* ethanol solution, after which they were treated with a solution of 4% NaOCl supplemented with Tween 80® (0.1% *v/v*) for five more minutes. Finally, the roots were washed with sterile double-distilled water for one minute and dried. Pieces of 4 × 0.5 cm or 1.5 × 0.5 cm were cut from the disinfected tissues under aseptic conditions and were placed in Potato Dextrose Agar (PDA, Difco® MD, USA) Petri dishes supplemented with 50 μg/mL of streptomycin (Str) and 50 μg/mL of amoxicillin (Am) to avoid bacterial growth.

The “leaf imprint proof” was used as a control to ensure the surface sterilization process was successful [23]. Briefly, the disinfected roots were pressed against a PDA Am/Str Petri dish and after one minute, they were retired; if no growth was observed, the surface disinfection process was considered to be effective. To further stress the endophyte character of the fungi, only the tips of those hyphae protruding from the roots were selected. Single hyphae tips were subcultured and transferred three times on PDA Amp/Str to ensure the purity of the isolates.

### 2.4. Identification of Fungal Strains

Fungal endophytes were identified by a biphasic method involving macro- and microscopic morphological observations of the conidia and hyphae, and through molecular DNA barcoding of the D1/D2 domain of large subunit (LSU-28S) rDNA, and to the Internal Transcribed Spacer (ITS) regions from the rDNA. The GenBank accession numbers of the LSU-28S sequences were assigned as MN900344 and MN900345 for the strains H17 and H21, respectively. For the ITS sequences, the GenBank accession numbers were MN900342 and MN900343 for strains H17 and H21, respectively.

Morphological analysis was carried out by growing the isolates in PDA and staining seven-day-old mycelia with lactophenol blue to observe under the microscope the asexual structures of reproduction such as conidiophores, conidiogenous cells, and conidia. For the generic level determination of two fungal strains, the monograph “The genera of Hyphomycetes” was consulted [24]. Other important taxonomic treatments were reviewed for confirmation of *Neocosmospora*, in particular *Fusarium solani* species complex (FSSC) [25,26,27,28,29] and *Aspergillus* [30,31,32] identification.

To perform the DNA barcoding analysis, first the fungal genomic DNA was extracted according to previous reports [33]. The quantity and quality of the DNA was verified by spectrophotometric measurements with an Epoch reader (BioTek instruments Inc, Vermont, USA) at 280/260 nm, and also through gel electrophoresis in 1% agarose Tris/Borate/EDTA buffer (TBE) gels.

To continue with the DNA barcoding analysis, the molecular marker sequences correspondent to D1/D2 domain and the ITS regions were amplified through PCR using the following primers: nu-SSU-0817-59 (5′TTAGCATGGAATAATRRAATAGGA3′), nu-SSU-1536-39 (5′ATTGCAATGCYCTATCCCCA3′), ITS1F (5′CTTGGTCATTTAGAGGAAGTAA3′), and LR21 (5′ACTTCAAGCGTTTCCCTTT3′), respectively. PCRs were performed with the following conditions: 30 cycles: 95 °C × 45 s, 55 °C × 45 s, 72 °C × 45 s and a final 10 minute run at 72 °C. Sequencing of the products was performed by the method of Sanger at the Unidad de Secuenciación of the Instituto de Biotecnología, UNAM. The phylogeny was inferred by using the SeaView platform Version 5.0 (Lyon, France) [34] following a Maximum Likelihood (ML) approach [35]. Homologous sequences were retrieved from material type nucleotide collections databases through BLAST search.

Manually cured individual gene alignment for each sequence was concatenated, and then analyzed together to infer the phylogeny. The concatenated DNA data matrix was submitted to SMS server in order to predict a suitable substitution model according to Akaike Information Criterion (AIC) [36]. Generalized Time Reversible model (GTR) model [35] + Gamma was selected as best-fitting model. Branch support was evaluated according to aLTR (SH-Like) algorithm [37]. Finally, the tree was graphically handled into FigTree version 1.4.4 (Edinburgh, UK) Java application for definitive presentation.

### 2.5. Tolerance Tests to Heavy Metals

According to previous studies [2], we decided to test zinc (Zn; ZnSO_4_·7H_2_O), lead (Pb; Pb(NO_3_)_2_), and copper (Cu; CuSO_4_·5H_2_O), since these heavy metals bioaccumulate in *V. farnesiana* leaves. To determine the tolerance of the endophyte isolates, we started by testing 100, 300, 500, 700, 1000, and 1400 parts per million (ppm) of Pb^+2^ and Cu^+2^, and 50, 150, 250, 350, 500, and 700 ppm for Zn^+2^, in PDA media at 28 °C for 8 days. To start the experiment, a 7 mm diameter cylinder of freshly grown mycelia in PDA without metals was inoculated in the center of the Petri dishes with the different metal concentrations, incubated at 28 °C, and the average growth diameter (AD) was measured with a ruler daily to determine metal tolerance [38]. Four perpendicular lines (L1–4) were drawn on Petri dishes, and the length of the diameter of the fungal colony was measured on each line for 8 days. The end of the experiment was considered when the fungal growth in the media without metals (control) filled the Petri dish (8 days). All measurements were carried out in triplicate. The average growth diameter was calculated according to the following equation: AD=L1+L2+L3+L4/4, (Equation (1)). The minimum inhibitory concentration was considered the one where no fungal growth was observed. The pH in the lead-containing Petri dishes was measured with pH test strips from Sigma Aldrich. When these data were obtained, we decided to measure fungal tolerance to the metal mixture (Pb, Cu, and Zn) to try to evaluate similar conditions of the Huautla mine tailing. Hence, we used several concentrations of each metal according to Supplementary Table S1.

### 2.6. Growth Rate and Inhibition of Growth

In each condition, the radial growth of the mycelia was measured daily in triplicate, means were plotted, and the slope was used to calculate the growth rate in cm per day. To calculate the percentage of growth inhibition, the average growth of the mycelial mat (in mm) was measured, taking the growth diameter of the control fungus as 100% and then subtracting it from the growth percentage of the fungi that were inhibited.

In the same manner, metals were also tested in combination, adding the amounts described in Table S1. The amounts of each metal in the combinations were selected from the data obtained from the single-metal growth experiments and are also mentioned in the footnotes.

### 2.7. Removal of Heavy Metals from Liquid Media

For these experiments, mycelia of the different strains were produced in Potato Dextrose Broth (PDB, Difco ® MD, USA) by growing them at 30 °C for up to 8 days (depending on the growth speed of the strain). Thirty grams of mycelium was inoculated in PDB in the presence (or absence as a control) of heavy metals (100 ppm of Cu; 100 ppm of Zn; or 350 ppm of Pb, each in separate tubes with fresh PDB or in a tube containing a mixture of 100 ppm of each metal). A two mL aliquot was taken from these cultures every 6 hours, centrifuged, and the supernatant was subjected to atomic absorption spectrophotometry for metal content, using an atomic absorption spectrophotometer (908AA, GBC Scientific Equipment Pty Ltd., GBC, Dandenong, Australia), with background correction. All the material was previously washed with ultrapure HNO_3_ (J.T. Baker ® NJ, USA) for 24 hrs. To ensure a satisfactory accuracy of the analysis, standard reference material of the National Institute of Technology and internal reference materials were used for precision, quality assurance, and control for selected metal measurements. For each measurement, average values of three replicates were recorded.

Metal content is reported as ppm (parts per million). Detection limits of the atomic absorption spectrophotometer are Zn: 0.0005mg/L, Pb: 0.01 mg/L, and Cu: 0.001 mg/L.

pH was measured at the end of the experiment.

### 2.8. Organic Acid Determination

Organic acids were determined by High-Performance Liquid Chromatography (HPLC Hitachi - Science & Technology, Berkshire, UK) using two kinds of methods. An isocratic method allowed us to detect fumaric, malic, succinic, and glutamic acids. It was performed in a Xbridge C-18, 3.5 µm (4.6 × 150 mm) column (Waters Corporation, Asse, Bélgica), and the mobile phase was phosphate buffer 0.01 M, pH 2.6, with a flow of 0.4 ml/min during 40 minutes. The injection volume was 10 µL, and the absorbance was read at 210 nm. A gradient method was also used in a ZORBAX NH_2_, 5 µm (4.6 × 250 mm) column (Agilent Technologies, Santa Clara, USA) with two mobile phases: 50% methanol and phosphate buffer 0.05M, pH 7, according to the conditions described in Supplementary Table S2. This method allowed us to detect glutamine, fumaric, malic, and succinic acids (Supplementary data). Measurements were performed in triplicate.

### 2.9. Extraction of Secondary Metabolites and TLC-Metabolic Profiling

The biomass from the liquid fungal cultures of *Neocosmospora* sp. (H17), grown in PDB with Pb (350 ppm) or without Pb salts, was filtered. Then, the extracts from the filtrate were obtained by liquid–liquid extraction using as separation solvent ethyl acetate (1:1 *v/v*). The organic phase was separated and dried at room temperature inside a fume hood. Afterwards, the dried extracts were protected from light and stored at 4 °C until use. The TLC analysis was performed according to previous reports [39]. Briefly, stock solutions of the extracts (50 mg/mL) and the reference standard boshaloside (iridoid) (3 mg/mL) were prepared. Then, bands of 0.5 cm (5 µL) of the extracts and standard were applied to TLC Silica gel 60 plates F254 (Merck ® Darmstadt, Germany) (8 × 4 cm). The plates were developed in a suitable mobile phase of chloroform:methanol (5:1 *v/v*) to separate the extracts. The TLC plates were first observed under visible light, UV-254 nm and 365 nm. Then, to detect the presence of secondary metabolites, the plates were derivatized with the following reagents: Ferric chloride 10% *w/v*, Sulphuric vanillin 0.1% *w/v*, Ninhydrin 2% *w/v* in ethanol, Borntrager’s reagent and its modified version, Dragendorff’s reagent, and Wagner’s reagent. The results of the metabolic profile of secondary metabolites were interpreted as described earlier [40,41]. The Retention fronts (Rf’s) of the separated bands were calculated and registered according to the following formula: Rf = distance travelled by the band / distance travelled by solvent front (Equation (2)).

### 2.10. Antioxidant Evaluation

#### 2.10.1. DPPH Bioautography

TLC Silica gel 60 plates F254 (Merck ® Darmstadt, Germany) were used for the analysis. The fungal extracts were applied in bands of 0.5 cm (5 µL) leaving 250 µg/spot, along with 30 µg/spot of the reference standards Boshnaloside, Quercetin, Resveratrol, Gallic acid, and Ellagic acid. After developing the plates in the mobile phase chloroform:methanol (5:1 *v/v*), they were dried and first observed under UV-visible light, 254 nm and 365 nm.

Next, the plates were derivatized with a methanol:water solution (80:20 *v/v*) of the free radical 1,1-Diphenyl-2-picrylhydrazyl (DPPH) (Sigma ® MO, USA) (0.08% *w/v*) and were incubated in darkness for 20 minutes [42]. The components with antioxidant activity were observed as bright yellow or cream color spots on a purple background, indicating their radical-scavenging capacity [40]. The Rf’s of the separated bands were calculated and registered.

#### 2.10.2. TEAC-DPPH Assay

The colorimetric properties of the fungal extracts were evaluated at a pH gradient from 2 to 14. Their maximum peaks of absorbance were determined through a spectrophotometric scan. The antioxidant activity by the DPPH method [43] was adapted for 96-well microplates. Double serial dilutions served to prepare the stock solutions of the fungal extracts (from 11 mg/mL to 0.077 mg/mL) using as diluent ethanol. Each well contained 200 µL of a methanol:water solution (80:20 *v/v*) of DPPH (125 µM) and 20 µL of the stock solutions of the fungal extracts to evaluate the final concentrations of 1 mg/mL to 0.003 mg/mL. Ethanol was used as a negative control. The tested concentrations were evaluated in quadruplicate. The plates were incubated for 30 min under darkness and read in a spectrophotometer at 515 nm. The results of antioxidant activity were expressed as % of DPPH discoloration and as TEAC (Trolox Equivalent Antioxidant Capacity) (µg Trolox/ mg of extract). A standard curve of Trolox served to compare the antioxidant capacity of the fungal extracts [44] (Supplementary data S2).

### 2.11. Statistical Analysis

All statistical analyses were performed with STATISTICA software version 8.0 (STAT Soft Inc. Tulsa, Oklahoma, USA). We use the Shapiro–Wilk “*W*” test, which is used to probe normality [45]. A Kruskal–Wallis nonparametric method was used since the data did not fit parametric models [45]. Subsequently a post hoc test of multiple comparisons was made to determine statistically significant differences between groups.

## 3. Results

### 3.1. Macroscopic and Microscopic Characterization of Fungal Strains from the Root of V. farnesiana

**Figure 1:**
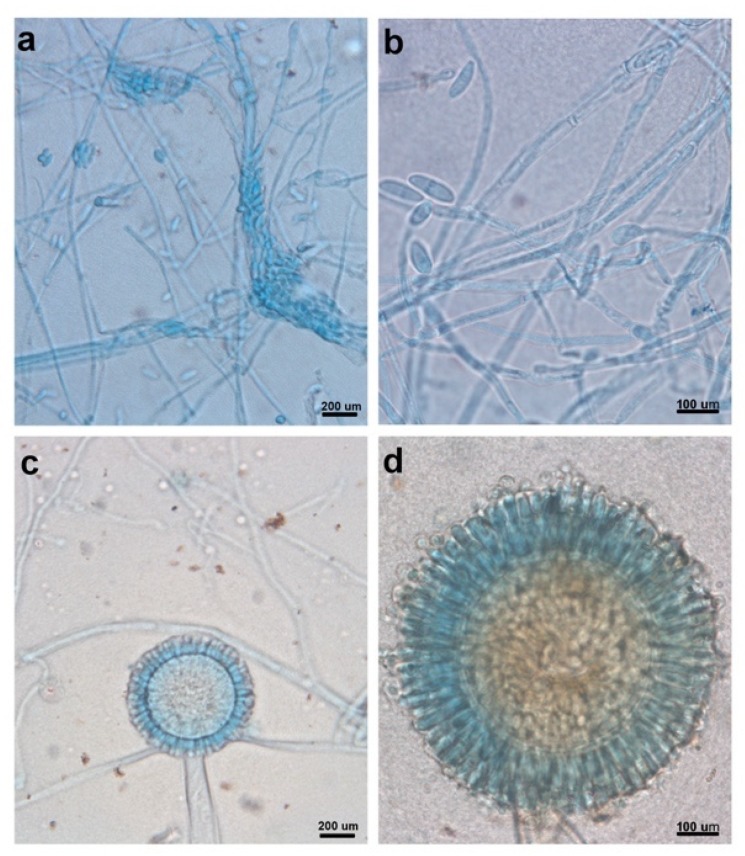
Microscopic observations of reproductive structures from strain H17 (**a** and **b**) and strain H21 (**c** and **d**). Scale bars are indicated accordingly (**a** and **c**: 200 µm; **b** and **d**: 100 µm).

Twenty-one fungal isolates were recovered from the roots of *V. farnesiana*, but most of them showed a very similar macroscopic appearance (Figure S1). Upon microscopic observation, we could see at least two clearly distinct morphologies (Figure 1). On the basis of preliminary tolerance experiments with 50 and 500 ppm of Pb, Zn, or Cu [46], we chose to further study the strains designated as H17 and H21 (one of each morphotype).

#### 3.1.1. Strain H17

The strain presented the following macromorphological and micromorphological characteristics:

Colonies in PDA grew rapidly, circularly with regular margins, and had a sparse, velvety, white mycelium; the reverse of the plate showed white mycelium with a smooth basal surface. Microscopic observations showed microconidia forming in the aerial mycelium from lateral phialides were hyaline, fusiform, and 1-septate (Figure 1a,b). Macroconidia were not observed Chlamydospores were found, being globose, smooth, and terminal or intercalary.

*Neocosmospora* is a pleoanamorphic genus and morphologically has been mainly identified by producing two types of conidia: (1) multiseptate, falciform, and pedicellate (with foot-shaped basal cell) macroconidia originating in monophialidic or sympodial poliphialidic conidiogenous cells that arise from a sporodochium and (2) mostly 0-1(-2) septate, oval, reniform, obovoid, pyriform, napiform, globose, and fusiform microconidia produced in monophialidic or poliphialidic conidiogenous cells that arise in the superficial mycelium [24,25,26,27,28]. Traditionally, the morphology, septation, and origin of macroconidia are the most important characters used in the identification of *Fusarium* species. Microconidia are not produced by all *Fusarium* species, so their presence alone is also a relevant character. The microconidia, the conidiogenous cell on which they are originated, and their disposition on and around the conidiogenous cell are all important diagnostic characters [28,29]. The study of the macromorphological characteristics and the morphology and ontogeny of microconidia suggest it is a fungus from the *Neocosmospora* genus.

**Figure 2:**
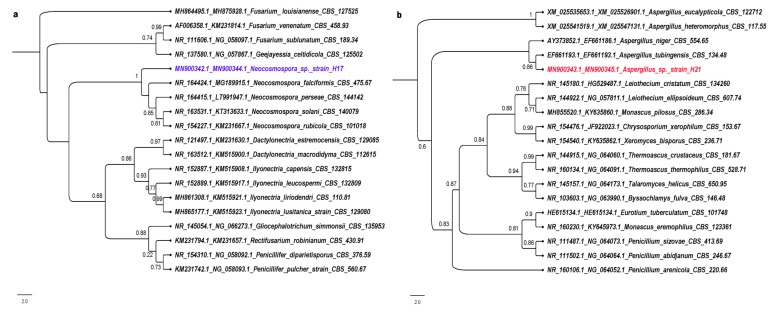
Phylogenetic reconstruction of the concatenated ITS1-ITS2–D1-D2 sequences for strain H17 (**a**) and H21 (**b**). Only sequences from type specimens and confirmed in publication were used for the phylogeny reconstruction. Accession numbers for ITS and D1-D2 regions (in this order) are shown for each specimen considered in the phylogeny.

Accordingly, phylogenetic analysis of concatenated ITS1-ITS2–D1-D2 ribosomal markers indicated that strain H17 belongs as a species within the *Neocosmospora* genus (Figure 2). Its closest relative is *Neocosmospora faciformis*. Based on the cladogram, we propose that strain H17 is a new species of *Neocosmospora*, since it is placed in its own distinctive branch with the highest support value. [47,48]. Although many of these species are plant and animal pathogens, the plant from which this strain was isolated did not show any signs of disease.

#### 3.1.2. Strain H21

This fungus presented the following macroscopic and microscopic characteristics:

Colonies in PDA with irregular shape and margins, convex, cottony, white, with a black mass of spores; the reverse of the plate showed a white mycelium with a smooth basal surface. Microscopic observations showed conidiophores consisting of thick-walled basal cells (foot cell), producing a long, aseptate, unbranched, smooth, and hyaline stalk that terminated in a globose vesicle. Also, conidiogenous phialidic cells were observed, borne on metulae circling the entire surface of the vesicle (conidial head biseriate). Conidia were present in dry chains, being globose and smooth (Figure 1c,d).

The condiophores of *Aspergillus* species are composed of a foot cell and an unbranched stipe, mostly without septa, which terminates in a vesicle. On the vesicle, the phialides can arise directly (uniseriate) or they are produced in whorls on metulae (biseriate). The conidia may be aggregated in columns or diverge in a radiating manner [30,31]. This particular morphology, named as aspergillum-like conidia-bearing structure, is the most important microscopic character traditionally used in defining members of this genus [49,50]. Nevertheless, it was found through phylogenetic analyses that the production of an aspergillum-like conidial head does not guarantee that a given species belongs to the *Aspergillus* genus. It has been also recognized that typical patterns of conidiophores are present in 90% or more of the species included in this genus, and the atypical patterns may occur in a minority of species, so the production of aspergillus-like conidial heads is essential for assigning a species to *Aspergillus* [49]. Regarding the infrageneric identification, there is no single method (morphological, physiological, or molecular) that works for the recognition of *Aspergillus* species. Therefore, a polyphasic approach should be used for species assignment, including morphological, physiological, and molecular data wherever possible [49,50].

According to morphological characteristics of the strain H21, mainly by the presence of an aspergillum-like conidia-bearing structure or aspergillum-like conidial head, together with the results of the molecular analysis, it can be asserted that this strain belongs to the *Aspergillus* genus, since the phylogenetic tree is positioned in a clade with several species of *Aspergillus*, being its closest relative *A. tubingensis* (Figure 2).

### 3.2. Tolerance to Heavy Metals

To evaluate the tolerance to heavy metals of strains H17 and H21, the fungi were grown in PDA with different concentrations of the metals, and the average growth diameter of the colony was measured daily.

**Figure 3:**
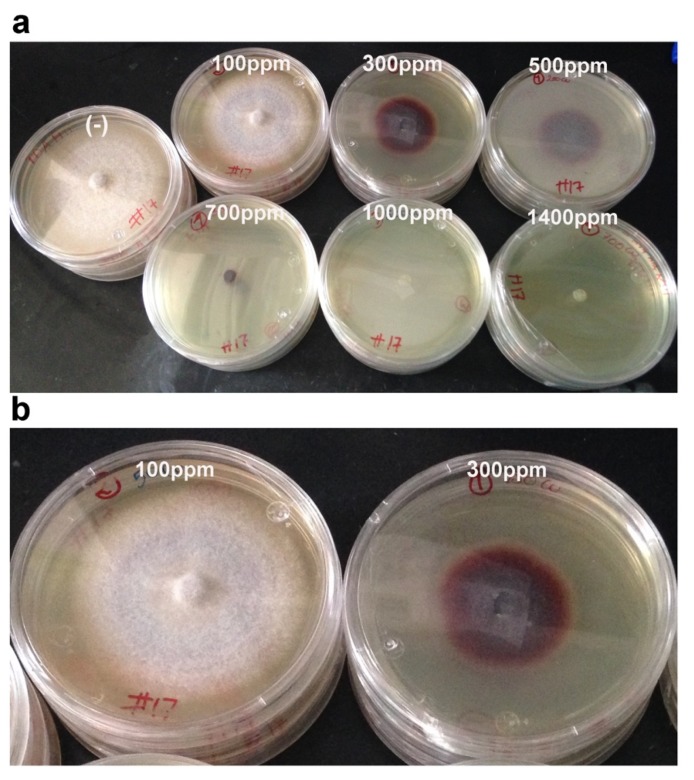
Growth of strain H17 in PDA with different Cu concentrations. (**a**) Copper concentrations tested for strain H17, a purplish pigment is produced as the Cu ppm increases, until no growth is achieved (1000 ppm). (**b**) A comparison between strain H17 grown in 100 ppm vs. growth in 300 ppm to stress the pigmentation in response to Cu. The picture was taken after 8 days of incubation at 28 °C.

Strain H17 could tolerate a maximum of 500 ppm of Cu, 700 ppm of Zn, and 700 ppm of Pb, with inhibition growth percentages of 55%, 86%, and 15%, respectively (Table 1). The best growth rate in the presence of the metal was obtained when we used the lowest concentration and was very similar to that one achieved in PDA (100 ppm for Cu, and identical, in fact, for Zn (50 ppm) and lead (100 ppm)). It is worth noting that this strain produced a purple pigment in response to Cu from the lowest concentration tested (100 ppm), which became more intense as the Cu concentration raised, until 700 ppm. Beyond that (1000 and 1400 ppm), no growth, and also no coloration, was observed (Figure 3). In PDA medium, the mycelium was white and did not show any kind of coloration; similar results were observed with lead (data not shown). Results using thin-layer chromatography suggest that this pigment could be related to phenolic compounds (see below).

**Figure 4:**
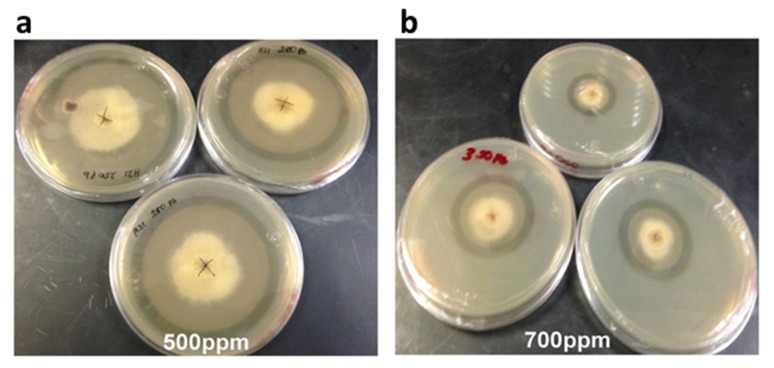
Growth of strain H21 in PDA with different concentrations of lead. (**a**) 500 ppm and (**b**) 700 ppm. Notice the translucent halo formed around the colony. Picture was taken after 8 days of incubation at 28 °C.

Strain H21 could tolerate a maximum of 1000 ppm of Cu, 350 ppm of Zn, and 700 ppm of Pb, with inhibition growth percentages of 88%, 97%, and 89%, respectively (Table 2). Its best growth rate was achieved again in the lowest concentration tested for each metal, except for Zn, where it was identical in 50 ppm as without the metal. For this strain, we noted a clear halo around the mycelial mat at 500 and 700 ppm of Pb (Figure 4). We measured the pH in this halo and observed that it dropped to 3, when the original PDA medium had 5.

We also tested heavy metals mixtures at different concentrations (supplementary Table S1). For strain H17, the growth rate was similar in most of the combinations tested and similar to the control without metals (around 0.98 cm/day vs. 0.9 cm/day for metal mixtures below 600 ppm), except in condition (e) (600 ppm Cu, 800 ppm Zn, and 700 ppm Pb), (f) (1000 ppm of each metal), and (g) (1000 ppm for Cu and 1400 ppm for Zn and Pb), where a growth rate decline was observed (Table 3). However, the growth inhibition was still not very significant in conditions (e) and (f) (22% and 49%, respectively), while in condition (g) a bigger decline was observed (around 66%). It is worth contrasting these results with those obtained for the same strain in single-metal media, since strain H17 still grows fairly well in condition (f) (42.9% growth inhibition; 1000 ppm for each metal) vs. its equivalent growth inhibition (around 50%), which in Cu is 500 ppm, in Zn is 250 ppm, and in Pb is above 700 ppm, so, the combination of metals seems to rise the threshold of tolerance.

The same was true for strain H21 (Table 4). When the metals were combined, this strain showed an increased tolerance to the amount of the three metals. For example, combination (i) had 700 ppm of Cu, 500 ppm of Zn, and 700 ppm of Pb, and growth inhibition was only 13.3%, while at the same single Cu concentration (500 ppm), growth inhibition reached 37%; more astonishingly, H21 tolerated 500 ppm of Zn in (i) but showed 50% growth inhibition at 150 ppm when this metal was alone. For Pb, the situation was similar, in combination with other metals growth inhibition was only 13% in (i), but in lead alone at 700 ppm, the fungus showed 89% of growth inhibition. In condition (j) (700 ppm of Zn and 1000 ppm of Pb and Cu), growth was severely impaired (74% of growth inhibition), and above 1000 ppm of Cu and 1400 ppm of Pb and Zn, no growth was observed (k) (Table 4).

### 3.3. Removal of Heavy Metals from Liquid Media

**Figure 5:**
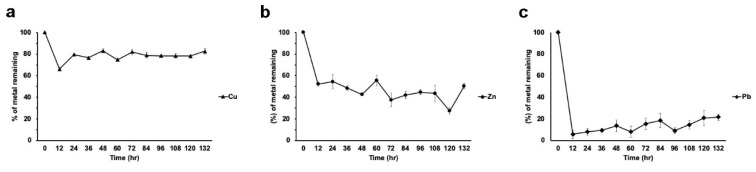
Metal removal from liquid cultures by strain H17. (**a**) Copper, (**b**) zinc, (**c**) lead. All treatments were tested in triplicate. Data is presented as the average amount of metal remaining in the media (%) ± standard deviation (error bars).

For these experiments, liquid medium with a concentration of 100 ppm of each metal was used. Strain H17 poorly removed Cu from the medium when it was the only metal present. Although at 12 hours a 34% removal was observed, in the subsequent samples we observed a desorption that returned almost to the original concentration levels (Figure 5a). However, the situation for Zn (100 ppm) was different. This metal was removed from the medium at 12 hours to 48% of the initial concentration and steadily decreased its concentration to around 27% at 120 h.; at 132 h, a desorption was observed to around 50% (Figure 5b). Strain H17 removed around 90% of the lead in the first 12 hours, and it slowly desorbed during growth, and at 132 h, around 30% remained in the medium (Figure 5c).

When the metal mixture was present in the medium, strain H17 presented a similar behavior with respect to metal removal, except for Cu, which in this condition was removed at the end of the experiment (132 h) to the same concentration as the other metals (between 20% and 30%, depending on the metal; see below, Figure 7a).

**Figure 6:**
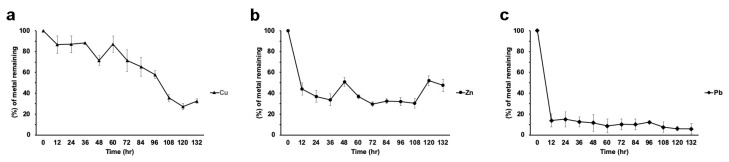
Metal removal from liquid cultures by strain H21. (**a**) Copper, (**b**) zinc, (**c**) lead. All treatments were tested in triplicate. Data is presented as the average amount of metal remaining in the media (%) ± standard deviation (error bars).

Strain H21 was able to remove up to 75% of the Cu in the medium when the metal was alone, (Figure 6a). Zn was rapidly removed by this strain (60% removal in 12 h; Figure 6b), although desorption of the metal was noted along the experiment, reaching at the end around 40% removal. Lead was removed to 15% of the initial concentration in the first 12 h and then decreased steadily, reaching about 8% of the initial concentration. (Figure 6c).

**Figure 7:**
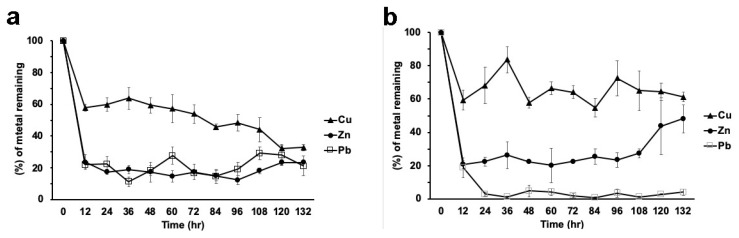
Metal removal from a mixture of metals (100 ppm each) in liquid cultures by strains H17 (**a**) and H21 (**b**). Data is presented as the average amount of metal remaining in the media (%) ± standard deviation (error bars).

However, when the three metals were present simultaneously, Cu showed an erratic kinetics of adsorption– desorption and it was removed to around 30%, from the medium at 132 h (Figure 7b). Zinc showed a rapid removal (of about 80%), but then desorption steadily increased, so at the end of the experiment, around 45% of the initial concentration remained in the medium. (Figure 7 b). Lead, however, was almost completely removed in the first 24 h and did not show desorption (Figure 7b).

### 3.4. Organic Acid Production in Response to Lead of Strain H21

Since strain H21 showed a halo around the colony when grown in the presence of lead and a significant pH drop was observed, the production of organic acids produced in response to lead by this strain was explored. Very clear differences were noted in the organic acid production in the presence or absence of lead. The most striking difference was the production of glutamic acid in the presence of the metal when no traces of this compound were found in the culture without lead (Table 5). Malic acid was increased around twelve-fold in the presence of lead, while succinic acid also showed an important increase of nearly four times. In contrast, fumaric acid diminished its concentration by fourteen-fold when the strain was grown in the presence of lead (Table 5).

### 3.5. Metabolite Profiling of the H17 Strain with or without Lead

**Figure 8:**
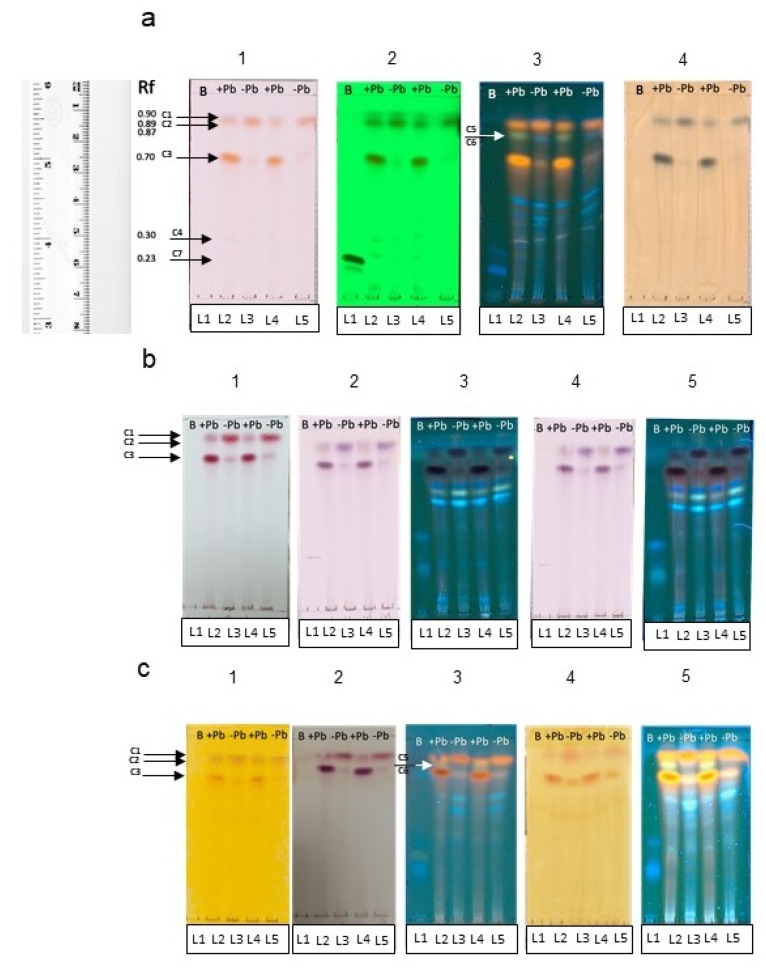
TLC analyses of secondary metabolites produced by strain H17 in presence (+Pb) or absence (-Pb) of lead. Boshnaloside (B) was used as reference standard. Retention front (Rf) of the compounds are indicated. (**a**) General derivatizers. (1) Visible light (Vis), (2) UV-254 nm, (3) UV-365 nm, (4) ferric chloride. (**b**) Detection of phenolics. (1) Sulphuric vainillin; (2) Borntrager-Vis; (3) Borntrager-365 nm; (4) modified Borntrager-Vis; (5) modified Borntrager-365 nm. (**c**) Detection of biogenic amines. (1) Draggendorff-Vis; (2) Ninhydrin; (3) Ninhydrin-365 nm; (4) Wagner-Vis; (5) Wagner-365 nm. Arrows (C1–C7) denote the bands that differentiate the chemical profile of both types of culture extracts.

At plain sight, fungal liquid cultures of *Neocosmospora* sp. (H17) presented variations in pigment production; cultures added with 350 ppm of Pb presented a purple coloration, while the cultures grown in basal medium were red (data not shown). After the liquid–liquid extraction, it was observed that most of the purple pigmentation found in the cultures added with Pb remained in the aqueous phase, while the ethyl acetate phase of both types of cultures remained with a red coloration. The TLC metabolic profiling of the ethyl acetate extracts from H17’s cultures with or without Pb enabled to detect differences in the secondary metabolite production patterns, particularly in the phenolic compounds group (Figure 8).

Both of the ethyl-acetate extracts from cultures with and without Pb contained a purple pigment observed under visible light in the Rf 0.90, seen in the lanes L2 and L4 (C1) (Figure 8a. panels 1–3) (quenched under 254 nm UV light and blue under 365 nm UV light). Particular differences were observed in the metabolic profile on both types of extracts, regarding the production of at least three phenolic compounds, whose bands were observed in the Rfs 0.89 (C2), 0.70 (C3), and 0.30 (C4), observed as reddish pigments without derivatization under visible light (Figure 8a panel 1), that quenched under 254 nm (Figure 8a. panel 2) and 365 nm UV lights (Figure 8a. panel 3), and observed as blue-green bands under visible light with FeCl_3_ (Figure 8a panel 4). On the basis of the observable color/quenching intensity and thickness of the bands, apparently the compound C3 (Figure 8a panels 1–4) is present at a higher concentration in cultures added with Pb, whereas C2 (Figure 8a panels 1–4) is present at a higher concentration in cultures grown in basal medium (Figure 8a panel 3). It was interesting to note that compound C4 (Figure 8a panels 1 and 2) was produced at a higher concentration in the cultures with Pb in comparison with the cultures without lead (Figure 8a panel 2).

The charring pattern of these compounds, observed with the different physical and chemical derivatizing agents to detect phenolics (Figure 8b panels 1–5), and the presence of biogenic amines or alkaloids (Figure 8c panels 1–5) indicated that C1 belongs to the group of alkaloids, while C2, C3, and C4 belong to the phenolic’s group, C2 and C3 are likely to be phenolic amines or anthocyanins, while C4 could be a tannin. None of the compounds belonged to anthraquinones or naphtoquinones according to the Borntrager’s test. It was also interesting to detect a difference in the fluorescence emission of two compounds (C5 and C6) with the same Rf of 0.87 (Figure 8a panel 3) (visible under 365 nm UV light) when supplemented with lead or not: in the extracts from the cultures added with lead, a light-green fluorescent compound is seen in the lanes 2 and 4 (C5), and a blue fluorescent compound (C6) was observed in the lanes 3 and 5 when the extracts from cultures without Pb were applied. The production of these compounds was also a characteristic difference between the two chemical profiles, and could indicate that the chemical skeleton of the compound produced in basal medium suffered a modification on its radical groups, and this was related to the presence of lead in the medium. Regarding the identity of these compounds, they could belong to the group of alkaloids or other biogenic amines, according to the results observed with the TLC derivatizing agents (Figure 8c panels 1–5).

Another difference in the chemical profile of the fungal cultures was given by compound (C7), seen as a band at Rf 0.23 (blue under 365 nm UV light and that quenched under 254 nm UV light) (Figure 8a panels 1–4). This compound was observed in the cultures added with lead, but not in the cultures grown in basal medium. Compound C7 is also related to the group of phenolics, and was also found to be produced differentially in cultures supplemented with lead.

**Figure 9:**
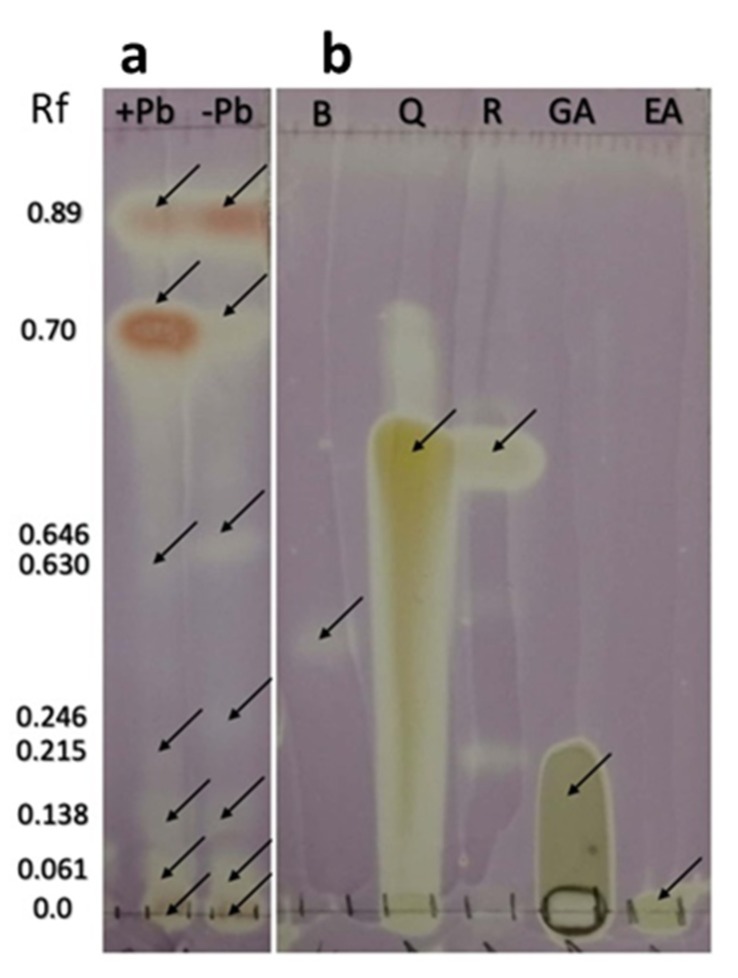
DPPH bioautography of strain H17’s extracts developed in chloroform:methanol (5:1 *v/v*). (**a**) Extracts from cultures in presence (+Pb) or absence (−Pb) of lead. (**b**) Reference standards: Boshnaloside (B), Quercetin (Q), Resveratrol (R), Gallic acid (GA), Ellagic acid (EA).

The TLC-bioautography with DPPH revealed that the extracts from the fungal cultures of H17 with and without Pb contained compounds with radical-scavenging activity (Figure 9). The compounds C2 and C3 (putative phenolic amines or anthocyanin-like compounds) described earlier reduced the free radical DPPH, forming a cream spot at their corresponding Rf. The presence of other compounds with antioxidant activity was evidenced at the bands found in the Rfs 0, 0.061, 0.138, 0.142, 0.215, 0.246, 0.630, and 0.646, which also reduced DPPH.

**Figure 10:**
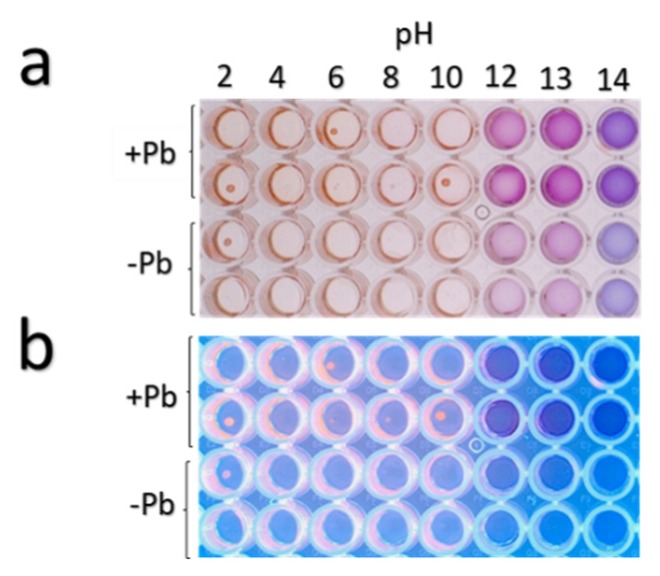
Changes in colorimetric properties of H17’s extracts from fungal cultures with (+Pb) or without (−Pb) lead under pH gradient. (**a**) Observed under visible light and (**b**) under UV light (365 nm).

As shown in Figure 10, the fungal extracts evaluated at a pH gradient (2–14) revealed that under a pH from 2–10 they presented red-pink color, whereas in a pH from 12–13 they presented a purple coloration, and at pH 14, the solution with the fungal extracts turned blue. The maximum peaks of absorbance of the fungal extracts subjected to the pH gradient were obtained as follows: red-pink coloration at pH 9–10 (λ_max1_ = 300 nm and λ_max2_ = 500 nm); purple coloration found at pH 12–13 (λ_max1_ = 290 nm and λ_max2_ = 555 nm), and blue coloration found at pH 14 (λ_max1_ = 295 nm and λ_max2_ = 580 nm). These changes of color in the presence of different pH solutions corroborated the presence of phenolic-amines or anthocyanin-like compounds [51]. Absorbance measurements indicated that the strain *Neocosmospora* sp. (H17) over-produces such compounds in the presence of lead in comparison with cultures grown in basal medium.

**Figure 11:**
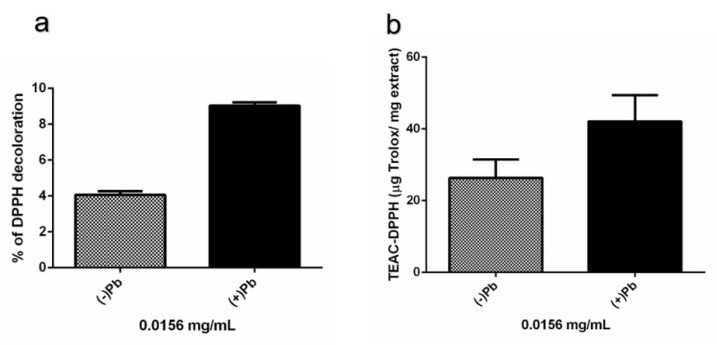
In vitro antioxidant activity of strain H17’s extracts from cultures in presence (+Pb) or absence of lead (−Pb). (**a**) Percentage of DPPH decoloration produced by the fungal extracts tested at 0.0156 mg/mL. (**b**) Trolox Equivalent Antioxidant Capacity (TEAC-DPPH) of the fungal extracts tested at 0.0156 mg/mL. Treatments were tested in quadruplicate. Data is presented as the average percentage of DPPH decoloration (%) and TEAC-DPPH (μg Trolox/mg extract) ± standard deviation (error bars).

Since maximum peaks of absorbance of the extracts at pH 2–10 were close to the recommended wavelength to carry out the antioxidant assay with TEAC-DPPH (515 nm), double serial dilutions of the extracts were used to assess the antioxidant activity testing concentrations where the color of the extracts did not interfere with the absorbance measurements. This quantitative evaluation revealed that at the concentration of 0.0156 mg/mL, the fungal extracts with and without Pb had an antioxidant effect by decoloring 9.04% and 4.06% of DPPH, respectively. Based on the extract’s percentages of DPPH discoloration, the extracts from cultures with and without lead had values of 42.09 and 26.34 µg Trolox/mg in terms of the TEAC-DPPH activity, respectively (Figure 11). These results indicated that the extracts from the fungal cultures grown in the presence of Pb had a higher level of antioxidant activity compared with the cultures without Pb.

## 4. Discussion

The role and definition of fungal endophytes are still unclear [52,53]. In this work, we selected *Vachellia farnesiana*, a metal hyperaccumulator plant with a wide geographic distribution, to study some of its fungal endophytes. The plant specimens were well established in the main Huautla’s mine tailing (Figure S3). This plant is commonly known in Mexico as “huizache”. It is a fast-growing shrub, tolerant and easily adaptable to drought, extreme pH, low levels of organic matter, and high temperatures, with a wide geographic distribution in arid and semiarid zones in Mexico [54,55]. Also, this species has been found in sites with high levels of heavy metals in soils [56]. We isolated and characterized two endophyte fungi from the roots of *Vachellia farnesiana*.

The fungi characterized in this work were identified as a species of *Neocomospora* sp. and an *Aspergillus* sp. (strain H21). Both strains could tolerate higher concentrations of Cu, Zn, and Pb (700 to 1000 ppm) than most of the fungi reported in literature ([8] and references shown below).

*Neocosmospora* spp. are ubiquitous soil fungi and frequently found associated with plant roots [50,57,58]. Most frequently they are reported as plant pathogens (or even human pathogens in the case of *N. falciformis*), but in this work, the plants from which H17 was isolated looked healthy and showed no symptoms of disease.

There are reports on how endophytes can interact with plants in distinct manners, and a fungus that in some circumstances could be a pathogen can become just a colonizer [59,60,61]. In fact, under these circumstances, the fungus may be participating in the removal of heavy metals from the soil in cooperation with the plant due to its capacity to remove high Zn and Pb concentrations and even Cu when they are mixed together. These interactions have been reported for other fungi as well [62,63].

Other studies demonstrated that the increased tolerance to Pb in an environmental isolate of *Neocosmospora solani* (Mart.) L. Lombard & Crous (syn. *Fusarium solani* (Mart.) Sacc.) occurs by binding Pb to the cell surface, thus interfering with the metal’s entry into the cell until reaching the saturation limit at the cell’s surface [64,65]. This phenomenon could explain why when either strain is grown in the mixture of metals the general tolerance is augmented (Table 3 and Table 4), since Pb would remain in the cell surface, preventing the entrance of Zn or Cu into the cell. This mechanism of resistance to Pb might also be involved in endophytic isolate H17, since the phenomena of sorption and later desorption of the metal was observed after 72 h. The desorption of the metals from the fungi in the root could then be translocated by the plant to the foliar level.

It is known that endophytic isolates from the genus *Neocosmospora* (FSSC complex) are considered as “creative fungi” since they produce secondary metabolites of industrial interest [66]. The differential production of red pigments by fungal cultures added with Pb could also be part of a sorption mechanism that sequesters the metals into the fungal cells, thus lowering the concentration of the available metal and its toxicity [64,65]. In our study, we identified that the red pigments produced by H17 cultures added with lead are phenolic compounds (putative phenolic amines or anthocyanin-like compounds) that possess antioxidant activity. This could indicate that the production of such red pigmented metabolites is also associated to a protective mechanism to cope with the oxidative stress caused by the process of lead’s sorption and later desorption, in order to keep the mycelium viable when lead is again bioavailable in the medium (as observed under in vitro conditions). Our findings also point out that this strain could help its host, *V. farnesiana*, to tolerate heavy metals not only by sequestering them through biosorption, but also by producing phenolic radical-scavenging secondary metabolites that assist the plant to cope and resist the oxidative stress caused by the presence of bioavailable heavy metals in the root (further confirmation of this hypothesis is needed). It would be interesting to investigate if the production of this type of secondary metabolite serves also to promote plant growth, as it has been reported in another metal-tolerant endophytic *Neocosmospora* isolate [67]. In addition, it is worth mentioning that not all the endophytic metal-tolerant isolates are capable of producing pigments in response to different growing conditions [67], and microorganisms that have this capacity are biotechnologically valuable as bio-indicators [68]. Therefore, the capacity of strain H17 to differentially produce the phenolic red pigments in the presence of Pb could serve as a bio-indicator to detect polluted areas with lead.

Strain H21 belongs to the *Aspergillus* genera and also showed a high capacity to remove Zn, Pb, and Cu, especially when the metals are mixed together, an interesting fact since metals are present as complex mixtures in natural conditions. As this was also true for strain H17, we hypothesize that when metals are together, the transporters may compete for their internalization, and mechanisms of bioadsorption are responsible for metal removal without affecting the fungal viability [63,64,65].

To the best of our knowledge, there are no reports of fungi secreting glutamic acid to cope with heavy metal toxicity. However, in plants, a set of peptides called phytochelatins, which share a γ-glutamil-cysteine-glycine moiety, are responsible for the chelation of many heavy metals (AsO^2−^, Cd^2+^, Ga^3+^, Zn^2+^, Hg^2+^, Pb^2+^, Ag+, Sb^3+^) [6]. Instead, malic, gluconic, acetic, citric, and oxalic acids have been described to solubilize heavy metals by different microorganisms [9,69]. These molecules, especially oxalic acid, have been reported to immobilize and detoxify Cd, Cu, Pb, and Zn in bacteria [69,70]. Glutamic acid is synthesized from α-ketoglutarate that comes from the Krebs Cycle, which, in turn, is indispensable for energy generation in aerobic conditions in microorganisms. Succinic acid, which was elevated four-fold in response to lead, also comes from the Krebs cycle, so the question arises of why this particular *Aspergillus* strain is using such precious carbon-rich molecules to cope with heavy metal toxicity (although malic acid was also augmented in the presence of lead (twelve-fold), no oxalic acid was detected). An attractive possibility is that being an endophyte, the fungus receives photosynthates from the plant, and this carbon input allows the use of glutamic acid, which is more soluble than oxalic or malic acids (8,570 mg/mL vs. 220 mg/mL or 1000 mg/mL, respectively). The dissociation constant for the α-carboxyl group of glutamic acid is 2.19, a value in between that of oxalic (1.4) and malic (3.51) acids, while the γ-carboxyl is similar to that of oxalic acid (4.25 vs. 4.4) but lower than that of malic acid (5.03). These properties probably make glutamic acid a more versatile chelator than the other shorter-chain organic acids such as oxalic or malic acids [71]. However, its key position in the nitrogen assimilation pathway probably requires an excess of carbon to be spared as a defense molecule against heavy metal toxicity.

The association of plant and fungi for bioremediation strategies of soils polluted with heavy metal mixtures is a viable and ecologically friendly way to restore mine tailing sites or soils with high heavy metal content.

## Figures and Tables

**Table 1:** Growth and growth percentage inhibition values for strain H17 cultivated in different metal concentrations.

	Strain H17								
Metal	Cu			Zn			Pb		
ppm	Growth rate ^1^	Growth inhibition ^2^	SD ^3^	Growth rate ^1^	Growth inhibition ^2^	SD ^3^	Growth rate ^1^	Growth inhibition ^2^	SD ^3^
PDA	0.8	(-)	± 0.017	0.8	(-)	± 0.017	0.8	(-)	± 0.017
50	(-)	(-)	(-)	0.8	5	± 0.030	(-)	(-)	(-)
100	0.7	13	± 0.017	(-)	(-)	(-)	0.8	4	± 0.010
150	(-)	(-)	(-)	0.6	22	± 0.007	(-)	(-)	(-)
250	(-)	(-)	(-)	0.4	49	± 0.053	(-)	(-)	(-)
300	0.4	50	± 0.013	(-)	(-)	(-)	0.7	11	± 0.011
350	(-)	(-)	(-)	0.3	65	± 0.017	(-)	(-)	(-)
500	0.4	55	± 0.037	0.2	79	± 0.015	0.7	14	± 0.007
700	0	100	± 0	0.1	86	± 0.01	0.7	15	± 0.010
1000	0	100	± 0	(-)	(-)	(-)	0	100	± 0
1400	0	100	± 0	(-)	(-)	(-)	0	100	± 0

(-): Not determined; (0): No growth. All treatments were tested in triplicate. Values showed the average growth rate (cm/day) ^1^, growth inhibition (%) ^2^, and ± standard deviation (SD )^3^. PDA: Potato Dextrose Agar.

**Table 2:** Growth and percentage inhibition values for strain H21 in different metal concentrations.

Strain H21									
Metal	Cu			Zn			Pb		
ppm	Growth rate	Growth inhibition	SD ^3^	Growth rate	Growth inhibition	SD ^3^	Growth rate	Growth inhibition	SD ^3^
PDA	0.8	(-)	± 0.04	0.8	(-)	± 0.04	0.8	(-)	± 0.04
50	(-)	(-)	(-)	0.8	5	± 0.178	(-)	(-)	(-)
100	0.7	7	± 0.057	(-)	(-)	(-)	0.6	30	± 0.016
150	(-)	(-)	(-)	0.4	47	± 0.043	(-)	(-)	(-)
250	(-)	(-)	(-)	0.03	95	± 0.015	(-)	(-)	(-)
300	0.6	21	± 0.058	(-)	(-)	(-)	0.5	41	± 0.050
350	(-)	(-)	(-)	0.02	97	± 0.002	(-)	(-)	(-)
500	0.6	30	± 0.018	0	100	± 0	0.3	68	± 0.035
700	0.5	37	± 0.058	0	100	± 0	0.1	89	± 0.033
1000	0.1	88	± 0.018	(-)	(-)	(-)	0	100	± 0
1400	0	100	± 0	(-)	(-)	(-)	0	100	± 0

(-): Not determined; (0): No growth. All treatments were tested in triplicate. Data is presented as the average growth rate (cm/day) ^1^, growth inhibition (%) ^2^, and ± standard deviation (SD) ^3^.

**Table 3:** Growth rate and percentage of growth inhibition of strain H17 in the presence of heavy metals combinations.

Strain H17								
		Combination of Metals (ppm of Each Metal) ^1^						
	PDA	a	b	c	d	e	f	g
Average growth rate (cm/day)	0.98	0.9	0.92	0.91	0.89	0.76	0.56	0.3
Standard deviation ^2^	± 0.017	± 0.018	± 0.047	± 0.022	± 0.005	± 0.029	± 0.017	± 0.013
Average growth inhibition (%)	0	4.1	6.1	7.1	9.2	22.4	42.9	66.3

^1^ Combination of metals: detailed in Supplementary Table S1. (**a**) Cu (100), Zn (250), and Pb (300); (**b**) Cu (200), Zn (350), and Pb (400); (**c**) Cu (300), Zn (500), and Pb (500); (**d**) Cu (500), Zn (700), and Pb (600); (**e**) Cu (600), Zn (800), and Pb (700); (**f**) Cu (700), Zn (1000), and Pb (1000 ppm); (**g**) Cu (1000), Zn (1400), and Pb (1400). ^2^ Refers to the growth rate. All treatments were tested in triplicate.

**Table 4:** Growth rate and percentage of growth inhibition of strain H21 in the presence of heavy metals combinations.

Strain H21					
		Combination of Metals (ppm of Each Metal) ^1^			
	PDA	h	i	j	k
Average growth rate (cm/day)	1.14	1.079	0.99	0.196	0
Standard deviation ^2^	± 0.04	± 0.018	± 0.035	± 0.032	± 0
Average growth inhibition (%)	0	5.4	13.2	74.2	100

^1^ Combination of metals: detailed in Supplementary Table S1. (**h**) Cu (500), Zn (350), and Pb (500); (**i**) Cu (700), Zn (500), Pb (700); (**j**) Zn (700), Pb (1000), and Cu (1000); (**k**) Cu (1000), Pb (1400), and Zn (1400). ^2^ Refers to the growth rate. All treatments were tested in triplicate.

**Table 5:** Organic acids produced by strain H21 in the presence or absence of lead in PDB (Potato Dextrose Broth) medium.

Organic Acid	Concentration (µg/mL)	
	without Pb	with Pb
Glutamic	ND	1927.9 ± 53.79
Malic	11.8 ± 0.82	143.1 ± 2.11
Fumaric	4565.6 ± 7.43	324.2 ± 17.57
Succinic	21.2 ± 0.96	89.6 ± 1.83

ND: nondetectable. In bold are shown the organic acids whose secretion was augmented in response to lead addition. Note that the secretion of Fumaric acid was diminished in response to lead. Measurements were performed in triplicate. Data is presented as the average amount of organic acids in the media (µg/mL) ± standard deviation.

## Supplementary Materials

The following are available online at https://www.mdpi.com/2076-2607/8/2/226/s1, Figure S1: Morphotypes of fungi isolated from *V. farnesiana.* Table S1: Combination of heavy metals in ppm for different treatments for strains H17 and H21; Table S2: HPLC gradient method for the detection of organic acids roots; Figure S2: TEAC-DPPH analysis of antioxidant activity. (a) Standard curve of Trolox. (b) Fungal extracts tested in DPPH assay. Figure S3. Specimens of *V. farnesiana* growing at the site of collection in the Huautla’s mine tailing.
