# Supplementary material for: Characterization of Fungal Endophytes Isolated from the Metal Hyperaccumulator Plant Vachellia farnesiana Growing in Mine Tailings
Source: Microorganisms. 2020 Feb 8;8(2):226. doi: 10.3390/microorganisms8020226 (PMC7074743; doi:10.3390/microorganisms8020226)
Supplement: Supplementary file 1 [file microorganisms-08-00226-s001.zip › Supplementary Material RCFV Corrected.docx]

Supplementary Material


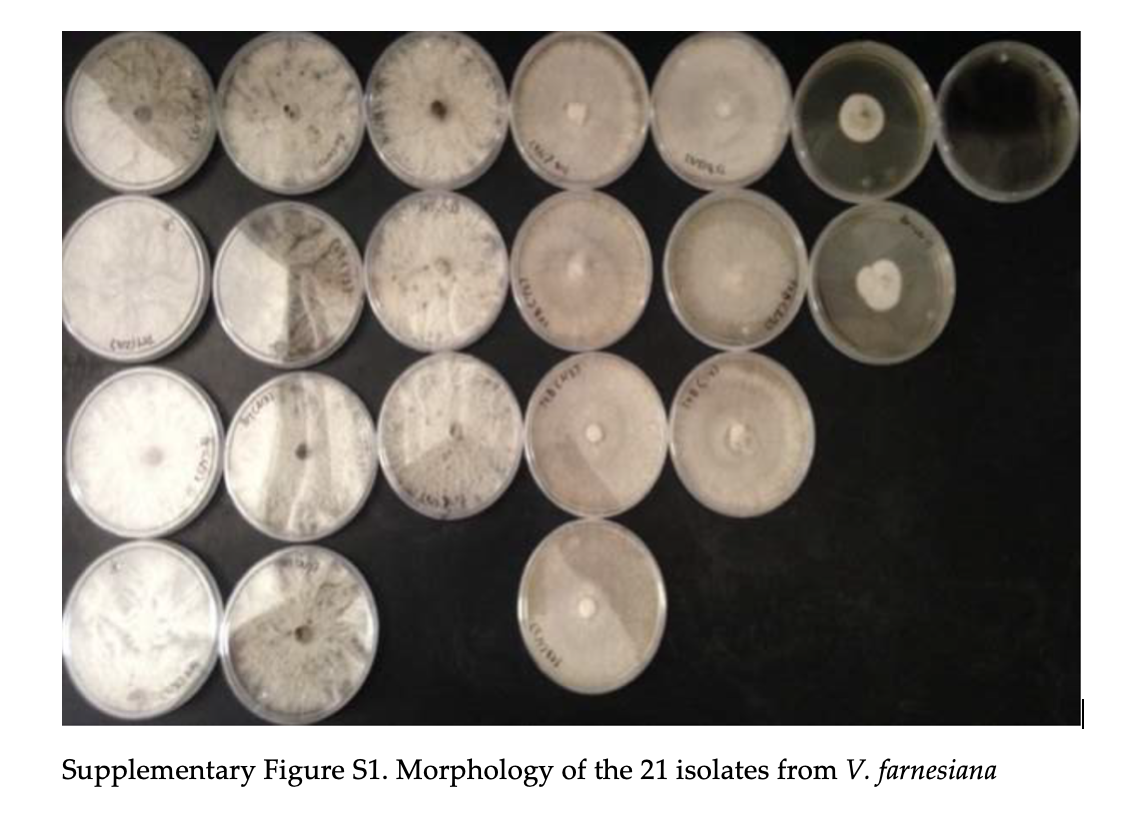


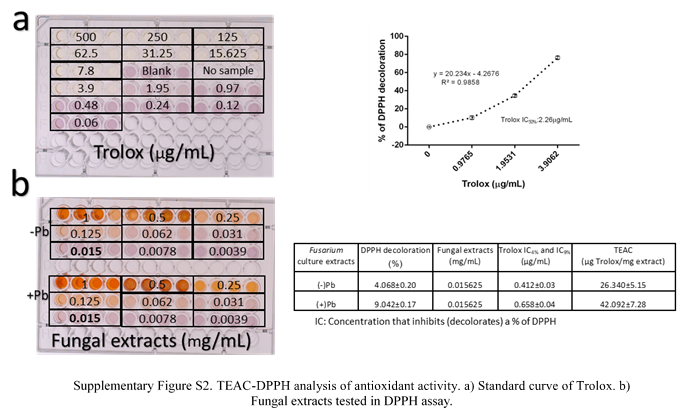


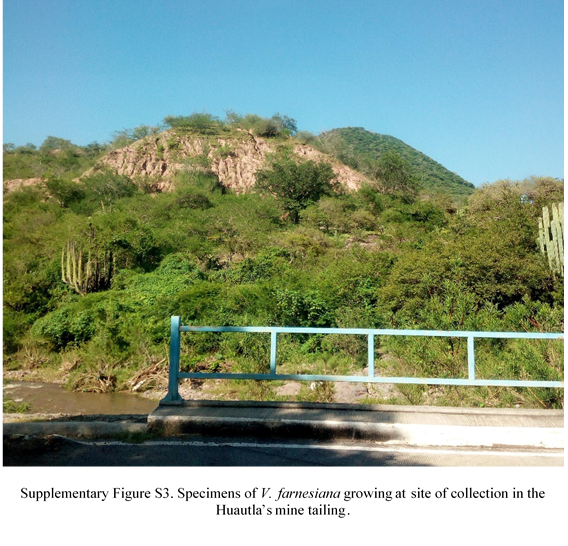


Supplementary Table 1. Combination of heavy metals in ppm for different treatments for strains H17 and H21

| H21 | | |
| --- | --- | --- |
| Treatment | Metal | Concentration |
| h | Cu | 500 |
|  | Zn | 350 |
|  | Pb | 500 |
| i | Cu | 700 |
|  | Zn | 500 |
|  | Pb | 700 |
| j | Cu | 1000 |
|  | Zn | 700 |
|  | Pb | 1000 |
| k | Cu | 1400 |
|  | Zn | 1000 |
|  | Pb | 1400 |

| H17 | | |
| --- | --- | --- |
| Treatment | Metal | Concentration |
| a | Cu | 100 |
|  | Zn | 250 |
|  | Pb | 300 |
| b | Cu | 200 |
|  | Zn | 350 |
|  | Pb | 400 |
| c | Cu | 300 |
|  | Zn | 500 |
|  | Pb | 500 |
| d | Cu | 500 |
|  | Zn | 700 |
|  | Pb | 600 |
| e | Cu | 600 |
|  | Zn | 800 |
|  | Pb | 700 |
| f | Cu | 700 |
|  | Zn | 1000 |
|  | Pb | 1000 |
| g | Cu | 1000 |
|  | Zn | 1400 |
|  | Pb | 1400 |

Supplementary Table 2. HPLC gradient method for the detection of organic acids

| Column | ZORBAX NH_2_, 5 µm  (4.6 x 250 mm) | | |
| --- | --- | --- | --- |
| Mobile phase A | Phosphate buffer 0.05 M, pH 7 | | |
| Mobile phase B | Methanol 50% | | |
| Flow | 0.6 ml/min | | |
| Time | Time (min) | Na_2_HPO_4_ | Metanol 50 % |
|  | 0 | 5 | 95 |
|  | 2 | 5 | 95 |
|  | 25 | 100 | 0 |
|  | 30 | 100 | 0 |
|  | 31 | 5 | 95 |
|  | 40 | 5 | 95 |
| Injetion volume | 10 µl | | |
| λ | 210 nm | | |
